# Successful control of vancomycin-resitant enterococci outbreak in a hematology unit

**DOI:** 10.1186/2047-2994-4-S1-P199

**Published:** 2015-06-16

**Authors:** H Jansens, F Van Laer, E Goovaerts, K Loens

**Affiliations:** 1 Infection Control, University Hospital Antwerp, Belgium; 2 Microbiology, University Hospital Antwerp, Belgium

## Introduction

Vancomycin-resistant enterococci (VRE) represent a major problem in healthcare settings worldwide. Controversies remain about the most efficacious infection control measures to reduce the rate of hospital spread.

## Objectives

To describe a recent VRE outbreak in our institution and the management of this outbreak.

## Methods

VRE was detected in clinical specimen by standard procedures from 3 different patients on a hematology unit. At that time, an infection control program was implemented. It included VRE screening in stool and rectal samples from all patients hospitalized in the epidemic ward and carriage screening to detect unknown colonized patients (at hospital admission, weekly and at discharge). Other infection control measures included: contact isolation of VRE carriers, daily cleaning and disinfection of the patient rooms and daily bathing of the VRE positive patients with antimicrobial washcloths. Cohorting of patients or staff was impossible due to full capacity of the hospital.

## Results

In total, from November 2014 to March 2015, 16 VRE cases were identified: 6 in clinical specimen (4 blood cultures) and 10 in screening samples. Following the identification of the 3 index cases, 9 additional patients were identified within the following 2 weeks, 8 by carriage screening and 1 in a clinical specimen. Two additional patients were identified on the intensive care unit (ICU); one was a transfer of a VRE positive patient of the hematology ward here to ICU with 1 additional transmission here. The same infection control measures were implemented in ICU and no extra transmission occurred. Two additional patients were found on the hematology unit 1 and 2 months after the beginning of the outbreak through the weekly screening samples. VRE isolates were identified as *Enterococcus faecium* carrying the vanA gen and molecular analysis showed that all isolates had the same Pulsed-field gel electrophoresis (PFGE) pattern. No newly acquired VRE colonization or infection occurred since February 2015.

## Conclusion

An outbreak of VRE on a hematology unit was controlled by daily bathing with antimicrobial washcloths in combination with reinforcement of environmental disinfection, both measures that reduce the microbial load.

## Disclosure of interest

None declared.

